# A Survey on Population Perceived Factors Influencing COVID-19 Vaccination in South Indian Districts

**DOI:** 10.7759/cureus.67696

**Published:** 2024-08-24

**Authors:** Raghav B, Jayakumar Rajagopal, Karthikeyan Ramaraju

**Affiliations:** 1 Medicine and Surgery, PSG Institute of Medical Sciences and Research, Coimbatore, IND; 2 Respiratory Medicine, PSG Institute of Medical Sciences and Research, Coimbatore, IND

**Keywords:** health communication, south indian districts, vaccine hesitancy, public perceptions, covid-19 vaccination uptake

## Abstract

The COVID-19 pandemic has posed unprecedented challenges to global public health, necessitating the rapid development and distribution of vaccines. Despite the availability of effective vaccines, vaccination uptake remains varied across different regions and populations. This study aims to investigate the factors influencing COVID-19 vaccination uptake in South Indian districts, with a focus on understanding public perceptions. Utilizing a cross-sectional survey methodology, data were collected from a representative sample of residents in selected South Indian districts. The survey explored a range of variables including demographic characteristics, knowledge and awareness about COVID-19 vaccines, perceived risks and benefits, trust in healthcare systems, and sources of vaccine-related information.

The preliminary analysis indicates that vaccine uptake is significantly influenced by factors such as age, educational level, and socioeconomic status. High levels of vaccine hesitancy were associated with misinformation, concerns about vaccine safety and efficacy, and distrust in government and healthcare authorities. Conversely, individuals with higher education levels and those who received accurate information from trusted sources showed a greater willingness to get vaccinated. Social and cultural beliefs also played a crucial role in influencing vaccination attitudes, stressing the need for the importance of culturally sensitive health communication strategies. The study investigates a range of factors, including demographic characteristics such as age, gender, education level, and socio-economic status, as well as psychological and social determinants like the perceived risk of COVID-19, trust in vaccines, and the influence of misinformation.

This study underscores the need for targeted public health interventions to address vaccine hesitancy and improve vaccination rates in South India. By identifying the key factors influencing vaccination decisions, policymakers and healthcare providers can develop more effective communication and outreach programs tailored to the unique needs and concerns of the population. Enhanced efforts in education, transparency, and community engagement are essential to fostering greater public trust and achieving higher vaccination coverage.

## Introduction

The COVID-19 pandemic has greatly impacted global health, economies, and daily life, prompting an urgent need for effective vaccines to mitigate the spread of the virus. Since their development, COVID-19 vaccines have proven to be a crucial tool in controlling the pandemic and reducing the severity of infections. However, despite the availability of vaccines, vaccination uptake varies significantly across different regions and populations. Understanding the factors that influence these variations is essential for improving vaccination rates and ensuring equitable health outcomes.

In India, a country with diverse cultures, languages, and socio-economic backgrounds, the factors influencing COVID-19 vaccination uptake are multifaceted. South Indian districts, known for their unique demographic and cultural characteristics, provide a compelling context for studying these factors. This study aims to explore the public perceptions and determinants of COVID-19 vaccination uptake in selected South Indian districts, offering insights into the complex interplay of influences that drive vaccination behavior.

Preliminary evidence suggests that vaccine hesitancy in South India is influenced by a combination of misinformation, cultural beliefs, and distrust in governmental and healthcare authorities. Conversely, higher education levels and access to accurate information from reliable sources correlate with increased willingness to get vaccinated. Understanding these dynamics is crucial for designing targeted interventions that address the specific barriers and facilitators of vaccination in this region [1]. Through a comprehensive examination of public perceptions and influencing factors, this study seeks to contribute to the broader effort of achieving higher vaccination coverage and safeguarding public health in South India [2].

## Materials and methods

Aim and objective

By using this comprehensive methodology, the study aimed to provide valuable insights into the factors affecting COVID-19 vaccination uptake in South Indian districts, ultimately contributing to more effective public health strategies [3]. This study employs a cross-sectional survey methodology to gather data from a representative sample of residents in South Indian districts. By analyzing this data, the research aims to identify key factors that influence vaccination decisions, thereby providing valuable insights for policymakers, healthcare providers, and public health professionals. The ultimate goal is to enhance vaccination uptake through tailored communication strategies, community engagement, and policy interventions that resonate with the local population's needs and concerns. The study also aimed to identify modifiable risk factors for vaccine hesitancy to inform future public health interventions.

Ethical considerations

Prior to commencing the study, approval was obtained from the institutional ethics committee of PSG Institute of Medical Sciences and Research. All participants were required to provide informed consent, ensuring they were fully aware of the study's purpose and their rights as volunteers.

Study location and duration

The survey was conducted at PSG Institute of Medical Sciences and Research, Coimbatore, in the year 2022. This institution was selected due to its diverse patient population and established a reputation for medical research.

Participant recruitment

A total of 574 volunteers participated in the study.

Inclusion criteria

Age

Participants aged 18 years and above were included in the study on factors influencing COVID-19 vaccination uptake in South Indian districts.

Residency

Residents of South Indian districts participated in the study on factors influencing COVID-19 vaccination uptake.

Willingness to Participate

Individuals consenting to join the study on factors influencing COVID-19 vaccination uptake in South Indian districts.

Language

Participants capable of comprehending and responding to the questionnaire in the specified language in the study on factors influencing COVID-19 vaccination uptake in South Indian districts. Findings highlighted linguistic accessibility as crucial, affecting perceptions of vaccination acceptance, misinformation awareness, and trust in healthcare systems.

Exclusion criteria

There were no specific exclusion criteria for this study. All eligible citizens who were willing to participate and could provide informed consent were included in the survey.

Data collection

Data were collected using a structured questionnaire, which participants could either fill out themselves or have completed with the assistance of an interviewer. The questionnaire was designed to be easily understandable by the general public, ensuring clarity and ease of response [4].

Questionnaire design

The questionnaire aimed to capture comprehensive data on several key aspects.

Impact of COVID-19

Participants were asked to assess the extent to which COVID-19 had affected their lives, including the severity of any infections they or their family members had experienced.

Vaccination Acceptance

Questions focused on participants' acceptance of the COVID-19 vaccine, their reasons for getting vaccinated or not, and any perceived barriers.

Side Effects

Information was gathered regarding any side effects experienced post-vaccination, providing insights into public concerns and experiences with the vaccine.

Feedback on Vaccination Services

Participants provided feedback on the services offered by vaccination centers, including the efficiency, accessibility, and overall experience.

Influence on Others

The survey also explored whether participants would encourage others to get vaccinated, assessing the potential for community-level influences on vaccination uptake.

Data analysis

The collected data were analyzed using IBM SPSS version 29 (IBM Corp., Armonk, NY) to identify the population-perceived factors influencing COVID-19 vaccination uptake. This included examining correlations between vaccination status and various demographic and clinical variables.

## Results

The demographic profile of the surveyed population revealed a diverse range of characteristics across various categories. In terms of age distribution, the majority of respondents fall within the 20-30 years bracket, constituting 60.1% (345) of the sample. Those aged between 31 and 40 years make up 32.8% (188), while individuals above 40 years constitute a smaller proportion at 7.1% (41) [5].

Gender distribution shows a notable majority of male respondents, comprising 66.6% (382) of the total sample, compared to 33.4% (192) who identify as female. This distribution indicates a slight skew towards male representation in the survey.

The majority, 65.3% (375), of vaccinations were administered to residents within the Coimbatore region, highlighting high local participation. Those residing within Tamil Nadu but outside Coimbatore accounted for 31.7% (182), indicating significant uptake across the state. Only 3% (17) of vaccinations were provided to individuals residing outside Tamil Nadu, underscoring limited participation from non-residents. This geographic distribution suggests localized efforts have been effective in promoting vaccination within the district and neighboring regions, emphasizing the importance of targeted outreach strategies to enhance vaccine accessibility and uptake.

Education levels among the respondents vary widely. A considerable 55.4% (318) have completed a university degree or diploma, reflecting higher education attainment within the surveyed group. 21.3% (12) have completed only school education, while 13.1% (75) have not progressed beyond primary school. A smaller yet notable 10.3% (59) have pursued a doctorate or professional education, indicating a segment of highly educated individuals in the sample.

Occupationally, the survey captures a diverse spectrum. A significant 56.3% (323) of respondents are employed in private or government sectors. Self-employed individuals or those engaged in business activities make up 22% (126), showcasing entrepreneurial and independent economic activities within the surveyed population. Retirement or unemployment is reported by 21.8% (125) of respondents, highlighting a segment outside the active workforce [6].

Overall, the data underscores a demographic snapshot that includes a predominantly young adult population, with a higher representation of males. Educational achievements are varied, with a notable portion having pursued higher education, and occupational diversity is evident across employment sectors and entrepreneurial endeavors. These findings provide valuable insights into the demographic composition of the surveyed population, offering a basis for further analysis and understanding of their characteristics and preferences.

**Table 1:** Demographic characteristics of study participants (N = 574).

Demographic variable	Categories	Frequency (n)	Percentage (%)
Age (years)	20-30 Years	345	60.1
	31-40 Years	188	32.8
	Above 40 Years	41	7.1
Gender	Male	382	66.6
	Female	192	33.4
Residence	Coimbatore region	375	65.3
	Within the state of Tamil Nadu (outside Coimbatore)	182	31.7
	Outside the state of Tamil Nadu	17	3
Education	Not attended beyond primary school education	75	13.1
	Completed school education only	122	21.3
	Completed university degree/diploma	318	55.4
	Doctorate/professional education	59	10.3
Occupation	Retired/unemployed	125	21.8
	Self-employed/business	126	22
	Employed with private/govt employers	323	56.3

The impact characteristics among the surveyed population reveal significant insights into their experiences and perceptions. When assessing the extent of life impact, the majority of respondents indicate varying degrees of influence. A notable 50.3% (289) report feeling somewhat impacted, while 25.1% (144) state they have been affected quite a lot. Conversely, 24.6% (141) feel they have not been impacted at all, reflecting a range of responses to life changes potentially influenced by external factors such as the COVID-19 pandemic [7].

In terms of the type of impact experienced, respondents report diverse effects across multiple dimensions. Financial impact emerges as the most prevalent, with 36.9% (212) of individuals indicating they have been affected financially. Psychologically, 33.1% (180) report experiencing impacts on their mental well-being, highlighting significant emotional consequences. Professionally, 27.5% (158) report effects on their work or career, underscoring disruptions within occupational spheres. A smaller proportion, 24.6% (141), claim they have not been affected in any specific way, suggesting varying degrees of resilience or insulation from external disruptions.

Regarding COVID-19 infection history, 27.0% (155) of respondents confirm having been infected, while 73.0% (419) either report no infection or are unsure of their status. This distribution provides insights into the prevalence of COVID-19 among the surveyed population, with a significant minority having directly experienced the virus.

Furthermore, the impact of serious illness or death among acquaintances is notably widespread, affecting 57.0% (327) of respondents. This statistic illustrates the broader societal implications of health crises, indicating that a substantial portion of the surveyed population has been affected by serious illness or death in their social circles.

Overall, these findings highlight the complex interplay of personal and societal factors impacting individuals within the surveyed population. From the varying degrees of life impact and types of influences experienced to the prevalence of COVID-19 infections and the ripple effects of serious illnesses or deaths among acquaintances, the data provides a nuanced understanding of the challenges and experiences faced by the respondents. Such insights are crucial for shaping targeted interventions and support measures to address the diverse needs arising from these impacts [8].

**Figure 1:**
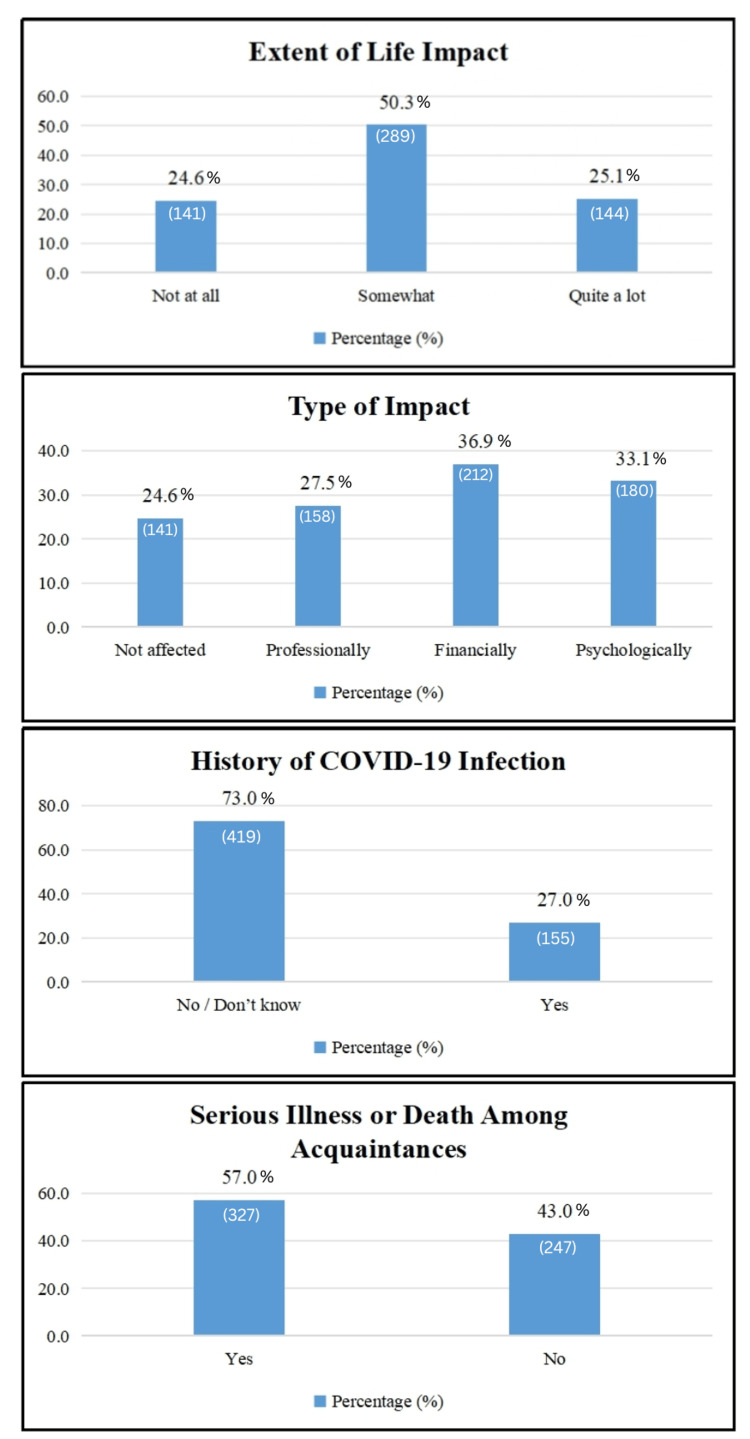
Impact of COVID-19 pandemic on the study population (N = 574).

The vaccination characteristics of the surveyed population provide valuable insights into their immunization status and post-vaccination experiences. Regarding the number of vaccine doses received, a significant majority have received the first booster dose, comprising 76% (436) of respondents. The second booster dose has been administered to 13.2% (76) of individuals, while 7% (40) have received only the initial dose. A smaller proportion, 3.8% (22), have not received any vaccine doses, highlighting varying stages of vaccination coverage within the surveyed group [9].

In terms of the type of vaccine received, Covishield emerges as the predominant choice, with 75.3% (432) of respondents opting for this vaccine. Covaxin follows at 19.5% (112), reflecting a significant but comparatively smaller proportion. Pfizer/Moderna and Sputnik vaccines are less commonly chosen, each accounting for 0.2% (one) and 0.7% (four) of respondents, respectively. This distribution underscores the prevalence of specific vaccines within the surveyed population, influenced by availability, accessibility, and individual choice.

Post-vaccination symptoms experienced by respondents showcase a range of reactions. A notable portion, 32.4% (179), report experiencing no symptoms following vaccination, indicating a lack of immediate adverse effects for a significant segment. Among those who did experience symptoms, fever is the most commonly reported, affecting 32.9% (182) of individuals. Body aches and tiredness follow closely, reported by 28.9% (60) and 23.5% (130) of respondents, respectively. Prolonged pain or local reactions at the injection site are reported by 18.6% (103), while headaches are noted by 17.4% (96) of individuals. These findings highlight the diverse physiological responses to vaccination among the surveyed population, emphasizing the variability in how individuals react to different vaccine formulations.

Overall, the data provide a comprehensive snapshot of vaccination trends, including coverage rates across different doses, preferences for specific vaccine types, and the spectrum of post-vaccination symptoms experienced. Such insights are crucial for monitoring vaccine uptake, understanding community health responses, and informing public health strategies aimed at enhancing immunization outcomes and addressing vaccine hesitancy or concerns [10].

**Figure 2:**
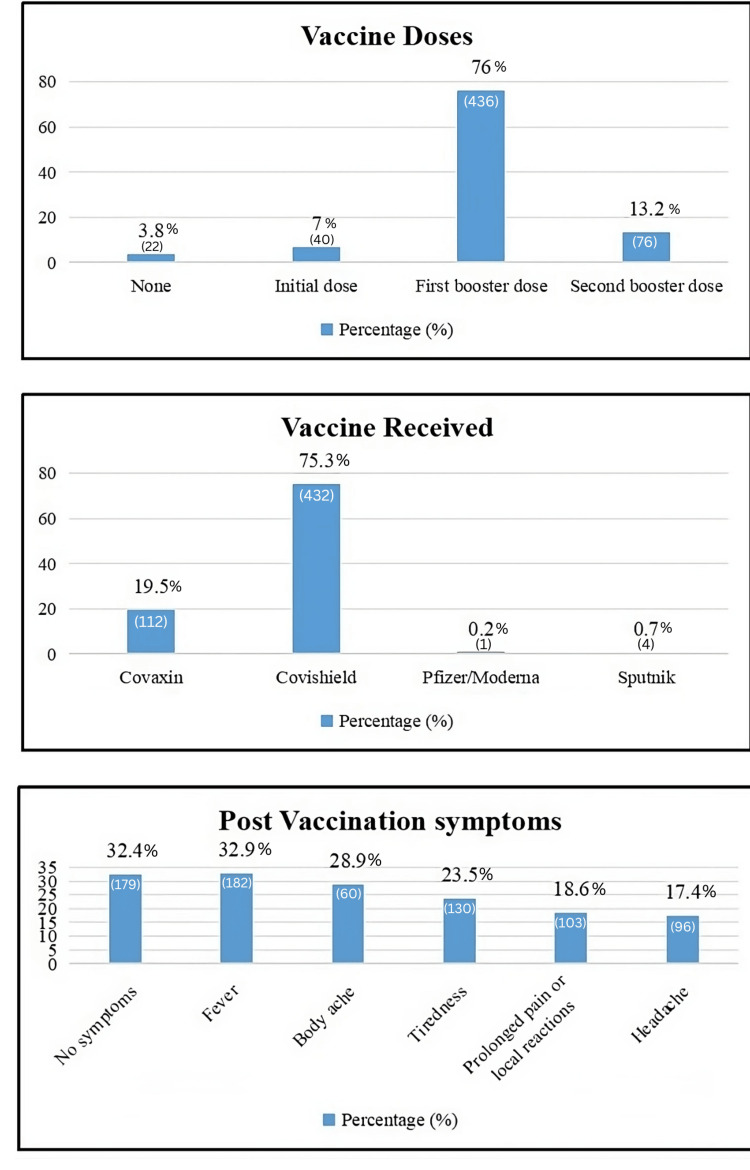
Vaccination characteristics of the study population (N = 574).

The reasons for vaccination among the surveyed population provide insights into their motivations and external influences driving immunization decisions. Compulsion from employers, both in government and private sectors, emerges as a significant factor, with 25.2% (129) of respondents indicating that employer mandates played a role in their decision to get vaccinated. This underscores the impact of workplace policies in influencing vaccination uptake [11].

The predominant reason cited for vaccination is personal health and safety, with 92.6% (474) of respondents stating this as their motivation. This reflects a strong individual commitment to protecting oneself from illness and maintaining overall well-being through vaccination.

Additionally, a smaller proportion, 7.8% (40), cites the need for a vaccination certificate as a reason for getting vaccinated. This requirement is often tied to accessing certain services or venues that mandate proof of vaccination status.

Compliance with travel regulations is another motivating factor, mentioned by 4.5% (23) of respondents. This indicates that travel-related requirements, such as international travel or entry to specific regions, influenced their decision to receive the vaccine.

Family compulsion is cited by 2.5% (13) of respondents, suggesting that familial expectations or concerns played a role in their vaccination decision-making process. Lastly, advisories on TV or social media influenced 3.5% (18) of respondents to get vaccinated. This reflects the impact of media and public health communications in shaping public perceptions and behaviors related to vaccination.

Overall, these findings highlight diverse motivations for vaccination, ranging from personal health considerations to external mandates and social influences. Understanding these reasons is crucial for designing targeted vaccination campaigns, addressing concerns, and promoting broader vaccine acceptance within the community [12].

**Figure 3:**
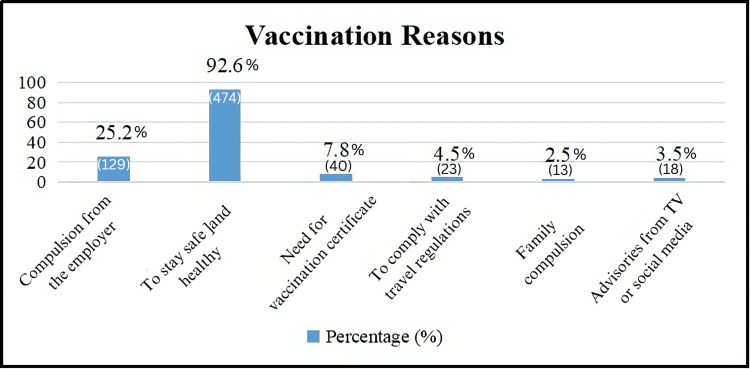
Reasons for vaccination (N = 512).

The reasons against vaccination among the surveyed population provide insights into the various concerns and factors influencing vaccine hesitancy or refusal within the community [13]. A notable proportion, 11.3% (seven), cites a lack of interest as a reason for not getting vaccinated. This indicates a segment of individuals who may not prioritize vaccination as part of their health practices. Fear of needle prick is mentioned by a small minority, accounting for 1.6% (one) of respondents. This fear can contribute to vaccine hesitancy among individuals sensitive to injections. Concerns about acquiring COVID-19 infection from the vaccine are expressed by 3.2% (two) of respondents. This fear reflects misinformation or misunderstanding regarding vaccine safety and efficacy. Confusion about vaccine types is cited by another 3.2% (two), indicating uncertainty or lack of clarity about different vaccine options available. Myths or rumors about vaccination are a significant factor influencing vaccine hesitancy, with 14.5% (nine) of respondents indicating they are scared by such misinformation. Addressing these myths is crucial for building trust and confidence in vaccination programs. Belief in traditional therapies is mentioned by 6.5% (four) of respondents as a reason against vaccination, reflecting cultural or personal beliefs that prioritize traditional remedies over modern medical interventions.

Background illness, either as advised by a doctor 12.9% (eight) or decided by the individual themselves 11.3% (seven), represents another significant barrier to vaccination. This indicates that pre-existing health conditions or medical advice play a role in vaccination decision-making for a notable segment of the population. Overall, these reasons against vaccination highlight a range of concerns and barriers, including misinformation, fear of side effects, cultural beliefs, and personal health considerations. Understanding these factors is essential for developing targeted interventions to address vaccine hesitancy, promote accurate information, and improve vaccination acceptance within the community [14].

**Figure 4:**
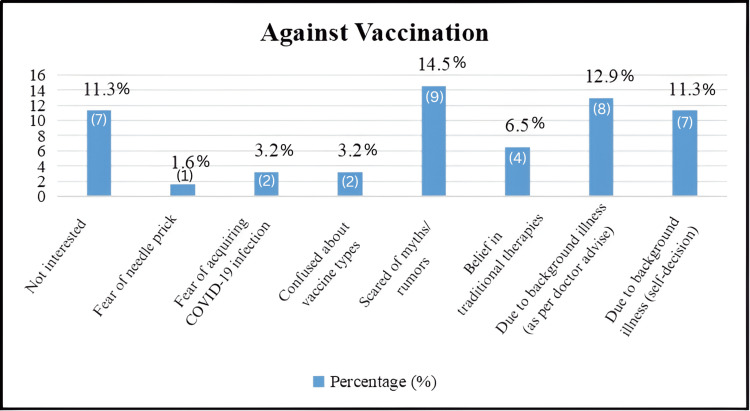
Reason against vaccination (N = 62).

The table presents the unadjusted odds ratios with corresponding 95% confidence intervals for factors potentially influencing a certain outcome, likely related to health, behavior, or another variable of interest [15]. Elderly age shows a statistically significant association with the outcome, with an odds ratio of 1.031 (95% CI 1.014 - 1.047). This suggests that for each additional year of age among elderly individuals, the odds of the outcome increase by approximately 3.1%.

Poor education level exhibits a strong association with the outcome, with an odds ratio of 2.96 (95% CI 1.59 - 5.50). This indicates that individuals with lower educational attainment have nearly three times higher odds of experiencing the outcome compared to those with higher education levels.

The presence of at least one co-morbid illness shows a significant association, with an odds ratio of 3.59 (95% CI 2.07 - 4.23). This suggests that individuals with one or more co-morbidities are over three and a half times more likely to experience the outcome compared to those without co-morbid conditions.

Financially affected during the pandemic exhibit an odds ratio of 1.61 (95% CI 0.95 - 2.73), which suggests a trend towards association with the outcome, although the confidence interval includes 1, indicating the association is not statistically significant at the conventional threshold of p < 0.05 [16].

Psychologically affected during the pandemic shows an odds ratio of 0.563 (95% CI 0.29 - 1.06). The confidence interval includes 1, indicating no statistically significant association between psychological impact during the pandemic and the outcome.

These findings highlight the varying degrees of association between different factors and the outcome of interest. Factors such as elderly age, poor education level, and the presence of co-morbid illnesses appear to significantly influence the outcome, while financial impact during the pandemic shows a weaker trend and psychological impact shows no significant association based on the provided data [17].

**Figure 5:**
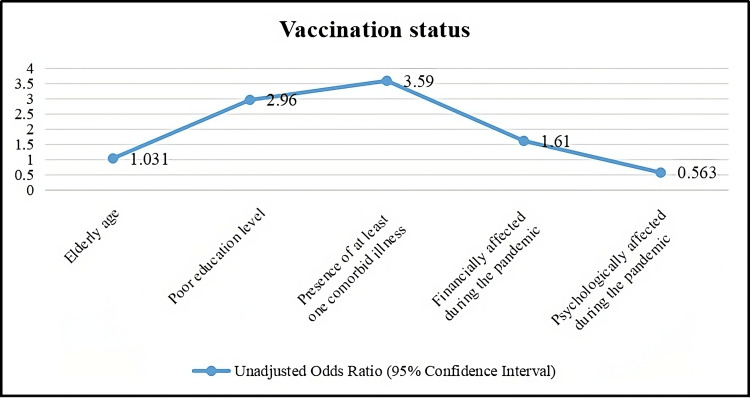
Factors associated with inadequate vaccination status.

## Discussion

The uptake of COVID-19 vaccination in South Indian districts is influenced by a myriad of factors, as reflected in the existing literature. A significant determinant is the level of public awareness and knowledge about the vaccine's safety and efficacy. Studies have consistently shown that misinformation and lack of accurate information can significantly hinder vaccination efforts. In South Indian contexts, community education and transparent communication from health authorities are paramount in addressing these gaps [18].

Cultural and religious beliefs also play a crucial role in shaping public perceptions towards vaccination. For instance, some communities may have reservations about the ingredients used in vaccines or concerns about potential side effects, which can be exacerbated by prevailing local myths and misconceptions. Engaging with community leaders and using culturally sensitive messaging can help mitigate these concerns and encourage higher vaccine acceptance [19].

Trust in healthcare systems and government institutions is another pivotal factor. Distrust, stemming from historical neglect or recent mishandling of health crises, can lead to vaccine hesitancy. Enhancing public trust requires consistent and empathetic engagement from healthcare providers and visible government commitment to addressing public health needs.

Socioeconomic status and accessibility also significantly impact vaccination rates. Individuals from lower socioeconomic backgrounds often face barriers such as lack of transportation, time constraints due to work, and insufficient healthcare facilities. Implementing mobile vaccination clinics and flexible hours can alleviate these barriers, making vaccines more accessible to underprivileged populations.

Insights revealed varied public perceptions impacting vaccination rates, highlighting crucial factors like accessibility, misinformation, and trust in healthcare systems. Findings underscored diverse public perceptions affecting vaccination rates, emphasizing accessibility challenges, misinformation impacts, and trust levels in local healthcare infrastructure. The research highlighted factors such as accessibility barriers, misinformation influence, and trust in healthcare services impacting vaccination acceptance among participants.

Lastly, peer influence and social networks are critical in shaping attitudes toward vaccination. Positive testimonials from friends and family members who have been vaccinated can greatly influence others to follow suit. Leveraging social media and local influencers to share positive vaccination experiences can therefore be an effective strategy [20].

In conclusion, improving COVID-19 vaccination uptake in South Indian districts requires a multifaceted approach addressing educational, cultural, trust-related, socioeconomic, and social network factors. Tailored interventions that consider these dimensions can enhance vaccine acceptance and uptake, contributing to better public health outcomes.

Limitations

Firstly, generalizability may be restricted due to the focus on a specific geographic area, potentially limiting applicability to broader populations. Additionally, the reliance on self-reported perceptions introduces the risk of response bias, where participants may provide socially desirable answers rather than their true opinions. Furthermore, the study's cross-sectional design may not capture changes in perceptions over time. Lastly, external factors such as media influence and political climate might not be fully accounted for, influencing vaccination attitudes independently of the studied factors.

## Conclusions

The study of public perceptions in South Indian districts reveals that COVID-19 vaccination uptake is influenced by multiple factors, including awareness, cultural beliefs, trust in healthcare systems, socioeconomic status, and peer influence. Addressing these elements through targeted community education, culturally sensitive communication, and improved healthcare accessibility can significantly enhance vaccination rates. Building public trust and leveraging social networks are also critical in overcoming vaccine hesitancy. Tailored interventions that consider these diverse factors are essential for increasing vaccine acceptance and achieving better public health outcomes in the region.
